# Cost-effectiveness analysis of the integrated control strategy for schistosomiasis japonica in a lake region of China: a case study

**DOI:** 10.1186/s40249-021-00863-y

**Published:** 2021-05-28

**Authors:** Ling-Ling Wu, He-Hua Hu, Xia Zhang, Xiao-Nong Zhou, Tie-Wu Jia, Can Wang, Zhong Hong, Jing Xu

**Affiliations:** 1 National Institute of Parasitic Diseases, Chinese Center for Disease Control and Prevention, Chinese Center for Tropical Diseases Research, NHC Key Laboratory of Parasite and Vector Biology, WHO Collaborating Centre for Tropical Diseases, National Center for International Research On Tropical Diseases, Shanghai, 200025 China; 2 Jiangling Station of Schistosomiasis Control, Hubei Province, Jiangling, 434100 China

**Keywords:** Schistosomiasis, Integrated control, Cost-effectiveness, Disability-adjusted life years, China

## Abstract

**Background:**

Schistosomiasis japonica remains an important public health concern due to its potential to cause severe outcomes and long-term sequelae. An integrated control strategy implemented in the Peoples’ Republic of China has been shown to be effective to control or interrupt the transmission of schistosomiasis. The objective of this study is to estimate the disease burden of schistosomiasis and assess the cost-effectiveness of the integrated control strategy focused on different major interventions at three stages for schistosomiasis control in a lake setting, to provide reference for policy making or planning.

**Methods:**

Annual cost data of schistosomiasis control during 2009–2019 were obtained from the control program implementers in Jiangling County, Hubei Province, China. Economic costs are provided in constant 2009 Chinese Yuan (CNY). Epidemiological data of schistosomiasis were collected from the Jiangling county station for schistosomiasis control. Disease burden of schistosomiasis was assessed by calculating years of life lost (YLLs) owing to premature death, years lived with disability (YLDs) and disability-adjusted life years (DALYs). DALYs were calculated as the sum of YLLs and YLDs. We then conducted a rudimentary cost-effectiveness analysis by determining the ratio by dividing the difference between the average cost of integrated control strategy at transmission control (2013–2016) or transmission interruption (2017–2019) and the average cost at stage of infection control (2009–2012) with the difference between the DALYs of schistosomiasis at different control stages. Descriptive statistics on the costs and DALYs were used in the analysis.

**Results:**

The total economic costs for schistosomiasis control in Jiangling County from 2009 to 2019 were approximately CNY 606.88 million. The average annual economic costs for schistosomiasis prevention and control at stages of infection control (2009–2012), transmission control (2013–2016), and transmission interruption (2017–2019) were approximately CNY 41.98 million, CNY 90.19 million and CNY 26.06 million respectively. The overall disease burden caused by schistosomiasis presented a downward trend. Meanwhile, the disease burden of advanced cases showed an upward trend with the DALY increased from 943.72 to 1031.59 person-years. Most disease burden occurred in the age group over 45 years old (especially the elderly over 60 years old). Taking the infection control stage as the control, the incremental cost-effectiveness ratio of integrated control strategy was CNY 8505.5 per case averted, CNY 60 131.6 per DALY decreased at transmission control stage and CNY −2217.6 per case averted, CNY −18 116.0 per DALY decreased at transmission interruption stage.

**Conclusions:**

The disease burden of schistosomiasis decreased significantly with the implementation of the integrated prevention and control strategy. Surveillance and management on elder population should be strengthened to decrease diseases burden. There remains a need for well-conducted studies that examine the long-term cost-effectiveness of the integrated control strategy for schistosomiasis.

**Graphic Abstarct:**

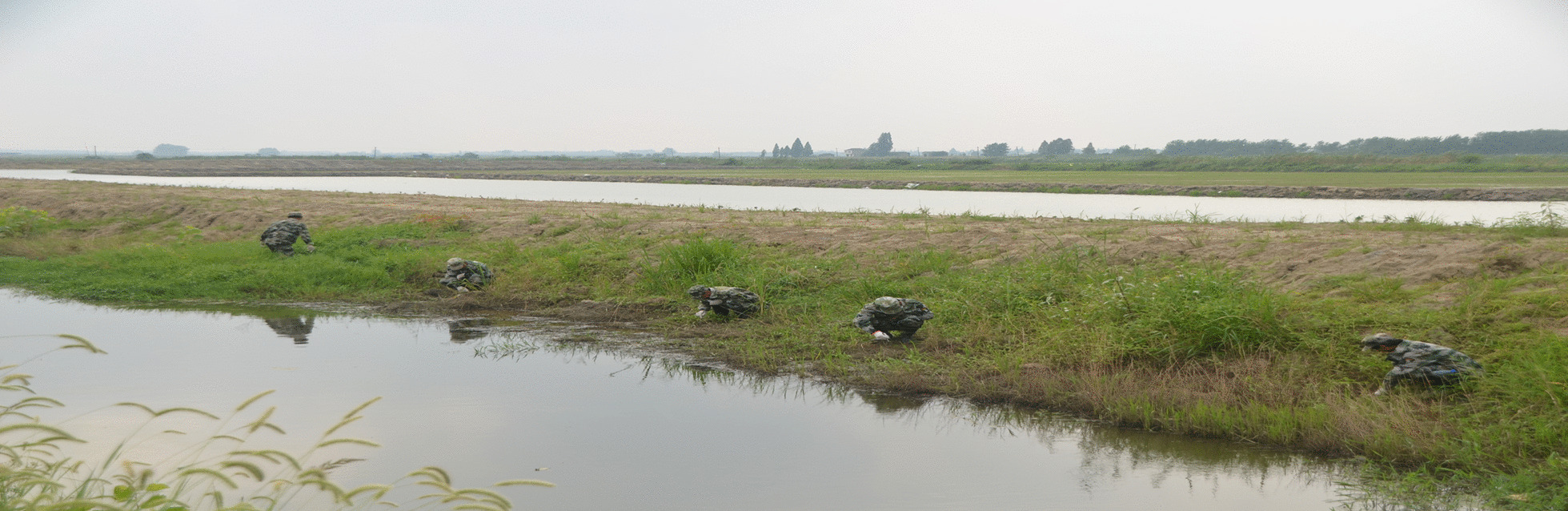

## Background

Schistosomiasis japonica is a zoonotic disease caused by trematode blood flukes of the genus *Schistosoma*, which infected 11.6 million cases and 1.2 million cattle in the People’s Republic of China (P. R. China) in the 1950s. [1] The implementation of an integrated control strategy focusing on the source of infection since the early stage of 21th century had strongly accelerated the process of schistosomiasis control in P. R. China [2]. Infection control (the infection rate of schistosomes in humans and livestock in epidemic villages < 5%) and transmission control (the infection rate of schistosomes in humans and livestock in epidemic villages < 1%) were reached nationwide in 2008 and 2015 respectively [3, 4]. By the end of 2019, 95.3% of the epidemic counties in P. R. China had reached the threshold of transmission interruption or elimination according to the national standards [5]. Currently, the key areas for schistosomiasis prevention and control in P. R. China are distributed along the middle and lower reaches of the Yangtze River, as well as the areas of the Dongting Lake and Poyang Lake. However, schistosomiasis japonica remains an important public health concern due to its potential to cause severe outcomes and long-term sequelae.

The integrated control strategy has shown to be effective to control or interrupt the transmission of schistosomiasis, especially in lake and marshland regions in P. R. China [6–8]. But disputation is ongoing: whether the investments should be continued as the prevalence decreased significantly in P. R. China, and whether this strategy could be transferred to other lower-middle-income countries with schistosomiasis as the integrated interventions were regarded as expensive. Being a component of health policy making and planning, conducting health economic evaluation could provide evidence to rationally allocate and utilize health resources and maximize benefits [9]. The health economics evaluation refers to the evaluation and selection of alternatives by analyzing the economic effects (cost, effect or input, output) of health planning [10]. Among the methods used to evaluate the economic value of disease interventions, cost-effectiveness analysis (CEA) is a routine one, which has been widely used [11, 12]. Although there have been several studies on the effectiveness of integrated prevention and control strategies for schistosomiasis, few are related to cost-effectiveness analyses [13].

As the integrated control strategy for schistosomiasis has been implemented in P. R. China more than 10 years, it is worthwhile to determine if this strategy is cost-effective as periodic assessment and analysis of resource utilization are conducive to the optimization of diseases prevention and control [14, 15]. The objective of this article is to understand the changes of disease burden due to schistosomiasis and evaluate the cost-effectiveness of the integrated control strategy focusing on blocking the transmission of schistosomiasis in a typical lake endemic region of schistosomiasis in P. R. China.

## Methods

### Survey setting

Jiangling County of Hubei Province, China, a typical lake region used to be a heavily endemic area of schistosomiasis, locates in the middle reaches of the Yangtze River and the north bank of the Jingjiang River. It belongs to the hinterland of the four lakes (Long Lake, Three Lakes, Egret Lake, Hong Lake), having rich water system and complex natural environments suitable for snails breeding. Through the implementation of the integrated control strategy for schistosomiasis, the county has reached the criteria of infection control, transmission control and transmission interruption in 2008, 2013 and 2017, respectively [16–18], keeping a low level of schistosomiasis endemicity until now.

### Data collection

From August to December in 2020, epidemiological data including the number of schistosomiasis cases (acute, chronic and advanced cases), area of snail habitats, the infected snails’ infested areas, etc. in Jiangling County from 2009 to 2019 were collected from the Jiangling County Station for schistosomiasis control, and used to assess the health effects of integrated control interventions. The general demographic information especially the age of each schistosomiasis cases were collected. Cost data related with schistosomiasis control in Jiangling County from 2009 to 2019 were obtained for the perspectives of program implementers.

### Determination of cost

The cost of the integrated control strategy for schistosomiasis includes expenditure of various control activities conducted by the department of health, agricultural, forestry, water conservancy, transportation and land modification etc. Cost data covered the costs of case detection and treatment, snail control, mollusciciding, health education, livestock disposal and management, toilets modification, risk monitoring, and other integrated interventions, etc [8, 19–21]. The costs of case detection and treatment were composed of the annual costs of questionnaire survey, blood examination, stool examination, treatment against schistosomiasis, involving the costs of subsidies, diagnostic reagents and materials, drug, medical assistance for advanced cases of schistosomiasis, etc. The costs of snail control included the costs for conducting snails’ survey and mollusciciding, involving the costs of subsidies, materials, and drug. Health education expenses included the costs for conducting training courses for school students, village cadres and doctors, the costs for preparing and distributing the leaflets, setting the warning signs. The costs of consumables and materials are the product of unit price and quantity. The costs of agricultural, forestry, water conservancy, transportation, land modification projects were directly collected from the related departments. The costs were presented in 2009 Chinese yuan (CNY) and a discount rate of 2.25% was used for cost.

### Health outcomes

Health outcome was presented in disability-adjusted life years (DALYs). DALYs were calculated by combining years lived with disability (YLDs) and years of life lost (YLLs). It was used to quantify the burden of disease, which was calculated with the life quality method based on disability weight (DW) got from cases’ life quality assessment [22]. The formula was listed as follows [23]:

$${\text{YLDs prev x }} = \sum Px *{\text{DWx}}$$

$$\mathrm{YLLs }=N*L$$

$${\text{DALYs}} = {\text{YLDs }} + {\text{YLLs}}$$

YLDs are the years lived with disability, YLDs prev x are the years lived with disability based on prevalence (prev is the abbreviation of prevalence, x is the grouping variable), Px is the number of cases in the reference population at a specific time. DWx is the disability weight of health outcomes. The DW of different age groups of advanced schistosomiasis cases was calculated as the following criteria: 30–44 years old (0.378), 45–59 years old (0.399), over 60 years old (0.510) [24]. The DW of different age groups of chronic cases of schistosomiasis was standardized as the follows: 5–14 years old (0.095), 15–44 years old (0.159), 45–59 years old (0.207), over 60 years old (0.246) [25].

YLLs reflecting life lost between the age of death and life expectancy at the age of death, are calculated by subtracting the age at death from the life expectancy at that age. For example, someone dying ages 56.5 at 2017 would lose 20.2 years of life when the life expectancy was 76.7 at 2017. The standard life expectancy was obtained from 2009 to 2019 life tables by year, gender and age offered by statistics bureau of local government.

### Incremental cost-effectiveness analysis (ICEA)

Incremental cost-effectiveness ratio (ICER) presents the additional benefit when extra money is invested to cross over from one intervention to another [26–28]. In this article, to analyze the cost-effectiveness of integrated control strategy with different major measures at different control stages, we used the ratio which was obtained by dividing the difference between the average cost of integrated control strategy at transmission control (2013–2016) or transmission interruption (2017–2019) and the average cost at stage of infection control (2009–2012) with the difference between the number of cases averted and DALYs of schistosomiasis at different stages.

## Results

### General information of schistosomiasis epidemic situation in Jiangling County

From 2009 to 2019, the endemicity of schistosomiasis in Jiangling County presented a downward trend. The number of clinical chronic schistosomiasis determined by serological tests and clinical symptoms dropped from 13 086 in 2009 to 2310 in 2019, while the number of confirmed chronic cases through finding *Schistosoma japonicum* eggs decreased from 2158 in 2009 to 0 in 2019. In addition, the number of advanced schistosomiasis cases dropped from 1248 in 2009 to 1073 in 2019, while no acute schistosomiasis case had been reported since 2008. The snail infested area had declined from 3030.73 hm^2^ in 2009 to 2718.04 hm^2^ in 2019, and no infected snail had been detected since 2012. (Table 1).

**Table 1:** Changes of schistosomiasis cases and snails’ infection status in Jiangling County from 2009 to 2019

Year	Disease situation			Snail situation	
	Number of clinical chronic cases	Number of determined chronic cases	Number of. advanced cases	Snail infested area (hm^2^)	Area with infected snails (hm^2^)
2009	13 086	2158	1248	3030.73	136.68
2010	11 264	1602	1192	3030.23	133.06
2011	9187	1199	1190	3029.53	51.51
2012	6988	573	1141	2875.86	0.00
2013	4682	395	1287	2875.86	0.00
2014	4455	229	1234	2875.86	0.00
2015	3251	74	1220	2728.65	0.00
2016	4003	0	1205	2728.65	0.00
2017	2852	0	1111	2718.10	0.00
2018	2330	0	1098	2718.04	0.00
2019	2310	0	1073	2718.04	0.00

### Costs of schistosomiasis control in Jiangling County

The overall expenditure for schistosomiasis control in Jiangling County from 2009 to 2019 was CNY 606.88 million, varied in the range of CNY 11.65–124.37 million annually. Classified by expenditure items, the costs from the land resources department for schistosomiasis control accounted for the largest proportion (about CNY 336.49 million, 55.45%), followed by costs of health activities for schistosomiasis control (about CNY 108.55 million, 17.89%). In the costs of health department, the costs of mollusciciding accounted for the highest proportion (CNY 38.26 million, 35.35%), followed by the costs of case treatment (CNY 24.66 million, 22.72%), while the costs of health education accounted for the lowest proportion (CNY 1.03 million, 0.95%). Calculating the costs by the disease control stage, the costs at the stage of infection control (2009–2012), transmission control (2013–2016), and transmission interruption (2017–2019) were approximate CNY 167.93 million, CNY 360.76 million and CNY 78.19 million respectively (Table 2). During the infection control stage, the costs of health control activity accounted for the highest proportion (52.31 million/167.93 million, 31.15%) of the total costs followed by the costs of transportation projects to modify the snail habitats (41.28 million/167.93 million, 24.58%). At transmission control and transmission interruption stage, the former two highest proportions of costs for schistosomiasis control were 78.00% (281.38 million/360.76 million), 44.15% (34.52 million/78.19 million) in the costs of land modification projects aiming to reduce snail breeding areas and 9.45% (34.10 million/360.76 million), 28.33% (22.15 million /78.19 million) in the costs of health control activities respectively.

**Table 2:** Costs of schistosomiasis control in Jiangling County from 2009 to 2019 (CNY 10 000)

Expense item		2009	2010	2011	2012	2013	2014	2015	2016	2017	2018	2019	Total
Special funds for health schistosomiasis control													
Costs of case searching		33.57	39.33	40.67	79.13	70.59	77.81	87.96	39.99	47.23	46.38	39.68	602.33
Costs of case treatment		255.47	244.07	229.69	217.77	217.14	226.21	230.58	236.73	213.54	199.90	195.04	2466.15
Costs of snails’ survey		37.64	39.18	43.42	33.44	38.90	33.31	79.46	80.35	67.59	54.03	54.00	561.32
Costs of mollusciciding		134.00	236.67	710.96	355.30	434.35	319.81	322.98	279.00	397.85	313.36	321.87	3826.15
Costs of health education		11.25	12.76	9.76	10.72	11.08	7.33	5.65	9.81	7.87	9.27	7.65	103.16
Costs of livestock disposal and management		132.00	1376.86	344.56	59.75	101.66	172.99	141.06	18.07	17.68	74.32	8.65	2447.60
Costs of toilets modification		200.01	283.66	0.00	0.00	0.00	0.00	0.00	0.00	0.00	0.00	0.00	483.67
Costs of risk monitoring		0.00	0.00	0.00	0.00	0.00	35.55	34.59	46.43	40.17	34.38	31.22	222.34
Others		12.25	21.03	12.96	12.64	17.18	11.52	10.81	11.40	13.85	9.82	9.16	142.62
Subtotal		816.18	2253.56	1392.02	768.75	890.90	884.53	913.08	721.78	805.78	741.46	667.27	10 855.32
Costs for integrated control													
Costs of agricultural projects		60.00	1334.28	478.24	187.09	0.00	304.19	321.58	175.76	217.14	128.64	102.53	3309.45
Costs of forestry projects		0.00	0.00	0.00	328.65	127.58	71.58	131.25	102.69	54.40	49.11	48.03	913.30
Costs of water conservancy projects		72.80	811.44	2104.34	0.00	0.00	501.04	420.01	299.52	242.71	342.14	256.16	5050.17
Costs of transportation projects		216.00	49.88	3098.98	763.31	768.47	232.63	455.01	616.15	150.65	70.39	489.91	6911.38
Costs of land modification projects		0.00	0.00	0.00	2057.94	4116.80	7985.31	10 195.78	5840.63	3452.37	0.00	0.00	33 648.82
Subtotal		348.80	2195.60	5681.55	3336.98	5012.85	9094.74	11 523.64	7034.75	4117.28	590.28	896.64	49 833.12
Total		1164.98	4449.16	7073.58	4105.74	5903.75	9979.27	12 436.72	7756.54	4923.05	1331.74	1563.90	60 688.43

The costs were presented in 2009 CNY and a discount rate was 2.25%

### Disease burden caused by schistosomiasis in Jiangling County

**Fig. 1:**
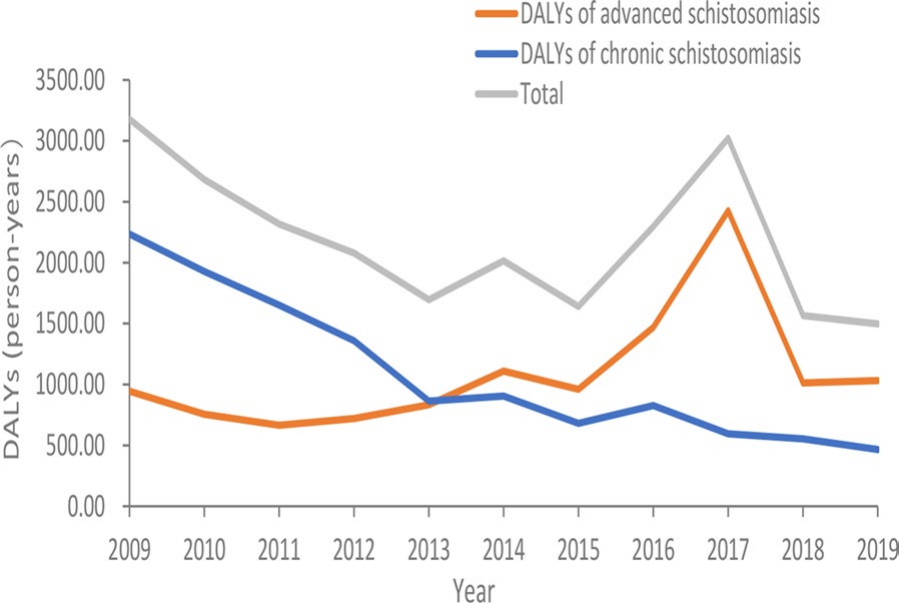
Changes of Disability adjusted life years caused by schistosomiasis in Jiangling County from 2009 to 2019

The overall disease burden caused by schistosomiasis presented a downward trend with DALYs decreased from 3178.38 person-years in 2009 to 1497.42 person-years in 2019. Calculating the disease burden by the types of schistosomiasis cases, the DALYs of chronic cases of schistosomiasis presented a downward trend (2234.66–465.83 person-years), while that of advanced cases showed an upward trend (943.72–1031.59 person-years) with a small peak in 2017. In addition, the variation trend in total DALYs of schistosomiasis was consistent with that of chronic cases before 2013, but shifted to be consistent with that of advanced cases after 2013. (Fig. 1).

### DALYs: disability adjusted life years

**Fig. 2:**
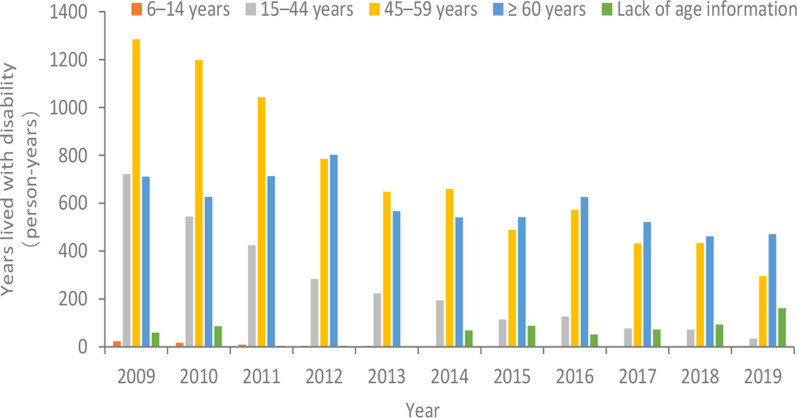
Changes of years lived with disability caused by schistosomiasis by age strata in Jiangling County from 2009 to 2019

According to DW, the schistosomiasis cases in Jiangling County from 2009 to 2019 were divided into 4 age groups: 6–14 years old, 15–44 years old, 45–59 years old and ≥ 60 years old. The YLDs in the 15–44 and 45–59 age groups presented an obvious downward trend (722.03–34.28, 1285.41–295.60 person-years), while fluctuated a little bit in the age group of 60 years old and above, in the range of 460 and 800 person-years. The annual YLDs was mainly concentrated in the age group of 60 years old and above, account-ed for 45.8% [43.9% (541.75/1233.77), 45.4% (625.43/1377.00), 47.2% (521.53/1103.92), 43.5% (461.47/1060.85), 49.0% (471.23/962.36)] on average during 2015 to 2019, while the YLDs in the age groups of 15–44 years old, under 15 years old accounted for 7.2% [9.3% (114.50/1233.77), 9.2% (126.16/1377.00), 7.0% (77.16/1103.92), 6.8% (71.85/1060.85), 3.6% (34.28/962.36] and 0.0% [0.0% (0.48/1233.77), 0.1% (0.76/1377.00), 0.0% (0.19/1103.92), 0.0% (0.29/1060.85), 0.0%] on average during 2015–2019 (Fig. 2).

### ICEA of integrated schistosomiasis control

During the research period, the number of schistosomiasis cases decreased from 10 897 at stage of infection control to 3717 at stage of transmission interruption. Taking the infection control stage as the control, we found that the integrated control strategy was more cost-effective (ICER: CNY – 2217.6 per case averted) in transmission interruption stage (Table 3).

**Table 3:** Cost-effectiveness analysis of integrated control strategy at different stages of schistosomiasis-number of cases

Stage of schistosomiasis control	Annual number of cases per year	Average number of cases averted per year	Average annual cost (CNY 10 000)	Incremental cost (CNY 10 000)	ICER(CNY)(incremental cost/average number of cases averted per year)
Infection control stage (2009–2012)	10 897	–	4198.4	–	–
Transmission control stage (2013–2016)	5229	5668	9019.1	4820.7	8505.5
Transmission interruption stage (2017–2019)	3717	7180	2606.2	−1592.1	−2217.6

– means the control, *ICER* incremental cost-effectiveness ratio, *CNY* Chinese yuan

Taking DALYs at a discount rate of 2.25% annually as the adjusted effectiveness indicator, we analyzed the ratio obtained by dividing the difference between the average cost at transmission control or transmission interruption and the average cost at stage of infection control with the difference between DALYs of schistosomiasis at corresponding stages. The ratio was CNY 60 131.6 per DALY decreased at transmission control stage and CNY −18 116.0 per DALY decreased at transmission interruption stage (Table 4).

**Table 4:** Cost-effectiveness analysis of integrated control strategies at different stages of schistosomiasis- disability adjusted life years

Stage of schistosomiasis control	Costs (CNY 10 000)	Increased costs (CNY 10 000)	DALYs (person-year)	Decreased DALYs (person-year)	Ratio(CNY) (increased cost /decreased DALY)
Infection control stage	16 793.5	–	9963.1	–	–
Transmission control stage	36 076.3	19 282.8	6756.3	3206.8	60 131.6
Transmission interruption stage	7818.7	−8974.8	5009.0	4954.0	−18 116.0

– means the control, *DALYs* disability adjusted life years, *CNY* Chinese yuan

## Discussion

The transmission of schistosomiasis is affected by biological, natural and social factors, thus the control of schistosomiasis is a systematic social project, involving many departments, such as agriculture, forestry, water conservancy, land resources, etc. Although the integrated control strategy focusing on the source of infection to block the transmission of schistosomiasis has been implemented in P. R. China more than ten years, it’s relatively difficult to carry out a systematic health economic evaluation on it. The economic evaluation indices of schistosomiasis control measures reported in previous studies were mainly the costs for reducing the infection rate of *S. japonicum* in residents, livestock, snails, or the costs for reducing the integrated impact index of schistosomiasis. [29–31] However, in current situation, the majority of schistosomiasis cases are advanced cases in P.R. China, featured with a long process of pathological changes and neglected impacts on residents' health. Thus previous evaluation indices were no longer applicable. In our study, the CEA of the integrated control strategy for schistosomiasis was conducted based on DALY, taking Jiangling County as an example, to explore the burden of schistosomiasis on population more scientifically and comprehensively.

The cost analysis of schistosomiasis in different periods in Jiangling County found that the average annual costs of schistosomiasis prevention and control at stage of infection control, transmission control and transmission interruption were approximately CNY 41.98 million, CNY 90.19 million and CNY 26.06 million respectively, equivalent to the annual per capita costs in schistosomiasis control of CNY 123.08, CNY 270.27 and CNY 77.29 respectively, if considering the number of average annual resident population (341 100, 333 700, and 337 200 in corresponding periods). Due to the large one-time costs in environmental improvement projects, the overall costs in schistosomiasis control were relatively high [32], but still reasonable from the perspective of population-based protection through prevention and control activities.

Advanced schistosomiasis cases were given healthcare priority due to its serious health and economic impacts, but it has not been included in the estimation of schistosomiasis burden in previous Global Burden of Disease (GBD) studies. In Jiangling County, there were only chronic and advanced schistosomiasis cases during 2009 to 2019 and the DW of schistosomiasis is seriously underestimated in GBD (0.005/0.006) [33, 34], thus we choose the age-specific DW cited from previous studies which aimed to estimate the age-specific disability weight of chronic and advanced schistosomiasis japonica in China based on the European quality of life questionnaire with an additional cognitive dimension (known as the “EQ-5D plus”) and participants’ self-rated health scores on the visual analogue scale of the questionnaire [24, 25]. In our research, the number of advanced schistosomiasis cases under 30 years old was 14 in 2009, with a mean value of 2.5 advanced cases annually in other years. Thus the advanced cases under 30 years old were directly included in the 30–44 age group. In addition, we took the mean value (0.177) as the DW of a few cases as their age information missed in this study.

The DALYs of chronic schistosomiasis was mainly based on YLDs caused by schistosomiasis rather than YLLs [35]. Our study found that the overall disease burden caused by schistosomiasis presented a downward trend. To the opposite, the disease burden of advanced cases showed an upward trend, which can be explained by the YLLs of advanced cases increased from 377.40 person-years in 2009 to 535.06 person-years in 2019. Also a small peak was presented in 2017 because 137 advanced cases were dead in that year (137 died cases), resulting in a larger YLLs. In addition, the current YLDs of schistosomiasis in Jiangling County mainly distributed in the age group of 60 years and over, followed by the age group of 45–59 years. The aging trend of cases was obvious, which was consistent with the existing researches [36, 37]. With the improvement of treatment quality and strengthening of medical assistance for advanced cases in P.R. China, the life expectancy of advanced cases will extend. As aging is an important factor affecting life quality and disease burden, it is necessary to strengthen dynamic monitoring and management of the elder age group through regular follow-up and healthy education. Early detection of advanced cases and timely interventions when the disease progression was found, are required to avert further deterioration leading to more disease burden.

ICER is often used to be an indicator to conduct cost-effectiveness analysis, represents the magnitude of additional health gained per additional unit of resources spent. And a country's gross domestic product per-capita was recommended by WHO as the cost-effectiveness thresholds. It should be noticed that in our study, the major interventions conducted in Jiangling County were adjusted with the decrease of prevalence of schistosomiasis, although the integrated control strategy for schistosomiasis was implemented since 2004. At infection control stage, key measures such as disposing of cattle or infected livestock, replacing cattle by machine for agriculture activities, improving sanitation, conducting transportation projects to modify snail breeding areas, etc. were supplemented to routine health interventions. At transmission control stage, measures of snail habitats modification in combination with utilization of land resource, preventing the rebound of feeding cattle, raising livestock in stall, case management were highlighted to decrease the infection of intermediate hosts and definitive hosts further. At transmission interruption stage, major tasks were adjusted to dealing with residual foci, identifying and managing the potential risks, surveillance and environmental modification to decrease the snail breeding areas. Thus we used the ratio by dividing the difference between the average cost of integrated control strategy at transmission control (2013–2016) or transmission interruption (2017–2019) and the average cost at stage of infection control (2009–2012) with the difference between the DALYs of schistosomiasis at different stages to analyze cost-effectiveness of integrated control strategy with different major interventions at different stages. Taking the infection control stage as the control, the ratio of integrated control strategy was CNY 8505.5 per case averted, CNY 60 131.6 per DALY decreased in transmission control stage and it was CNY −2217.6 per case averted, CNY −21 508.6 per DALY decreased in transmission interruption stage, suggesting that the strategy was more cost-effective in the transmission interruption stage. However, further studies should be conducted to explore the impact of various interventions on schistosomiasis control through deeper analysis.

There are some limitations of our study. First, we conducted the cost-effectiveness analysis from healthcare perspective, thus the costs of human resources and fixed equipment, were not included in the cost calculation due to its complicity of definition, which may impact on the findings. It is suggested to strengthen the recording and archiving of relevant data in the future in order to improve the quality of similar research thus to better guide on-site prevention and control. Second, being a rudimentary CEA, our research was based on observed data and we didn’t consider the impact of costs for future. Further studies based on modelling need be explored to better evaluate the future effect of the integrated control strategy for schistosomiasis japonica.

## Conclusions

The costs were relatively high at the early stage of schistosomiasis control, but the disease burden decreased significantly due to the implementation of the integrated prevention and control strategy. The study showed that the costs in schistosomiasis control decreased significantly after Jiangling reached the threshold of transmission interruption. The rising prices of materials and labors may further deepen the gap between investment and actual demand of schistosomiasis control. And, we find that the integrate control strategy is more cost-effective at the transmission interruption stage. Thus, multiple section collaboration and resource integration should be strengthened further and investments in the schistosomiasis integrated prevention and control should be continued to facilitate the elimination of schistosomiasis. In addition, the current disease burden of schistosomiasis mainly distributed in the elder age group. Early detection of advanced cases and timely interventions when the disease progression was found, are required to avert further deterioration leading to more disease burden.

## Data Availability

All the relevant data generated during this study are included in the manuscript.

## Abbreviations

CEA: Cost-effectiveness analysis
CUA: Cost-utility analysis
CBA: Cost–benefit analysis
CMA: Cost-minimization analysis
DALY: Disability adjusted life years
DW: Disability weight
YLDs: Years lived with disability
YLLs: Years of life lost
ICEA: Incremental cost-effectiveness analysis
ICER: Incremental cost-effectiveness ratio
*S. japonicum*: *Schistosoma japonicum*
P.R. China: People’s Republic of China
CNY: Chinese yuan
